# Supplementary material for: Decoding Natural Sounds in Early “Visual” Cortex of Congenitally Blind Individuals
Source: Curr Biol. 2020 Aug 3;30(15):3039–3044.e2. doi: 10.1016/j.cub.2020.05.071 (PMC7416107; doi:10.1016/j.cub.2020.05.071)
Supplement: Document S1. Figures S1–S4 and Tables S1 and S2 [file mmc1.pdf]

Current Biology, Volume *30*

Supplemental Information

Decoding Natural Sounds in Early "Visual" Cortex of Congenitally Blind Individuals

Petra Vetter, Łukasz Bola, Lior Reich, Matthew Bennett, Lars Muckli, and Amir Amedi

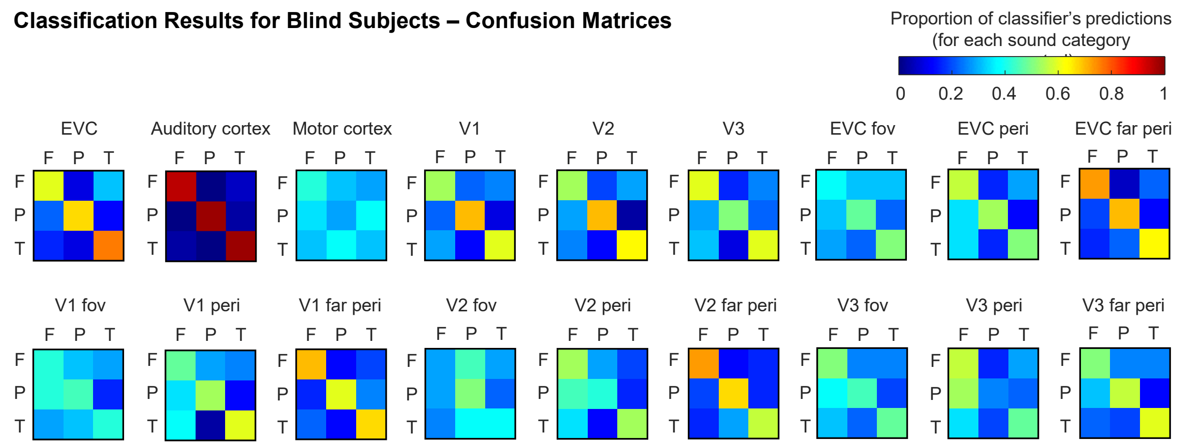

Figure S1. Confusion matrices representing classifier predictions for each sound condition in the group of blind participants. Related to Figure 1. Columns of the confusion matrices indicate the sound displayed (F, forest; P, people; T, traffic), and rows indicate classifier's predictions for this sound. The classifier's predictions for each sound are represented by colour hues, with warmer colours for higher proportion of predictions and colder colours for lower proportion of predictions. Note that in the ROIs in which above-chance classification accuracy was observed, the classifier was generally able to differentiate between all three sounds. EVC – early visual cortex.

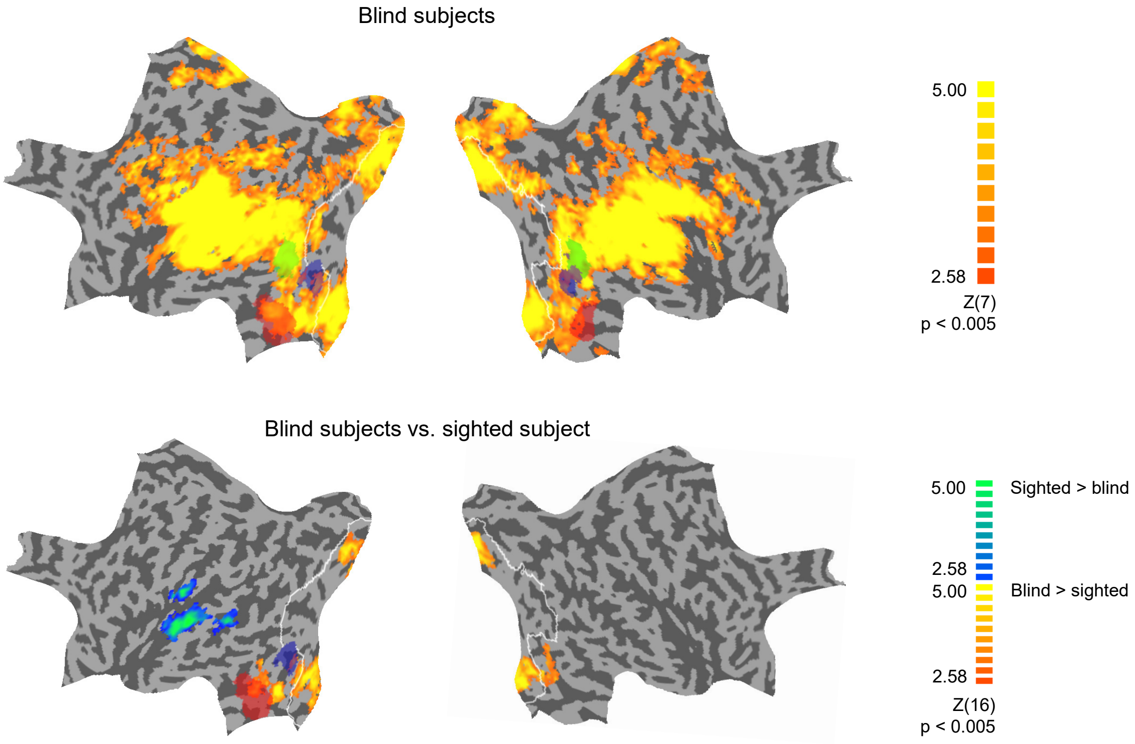

Figure S2. Results of the whole-brain searchlight analysis within a non-parametric model. Related to Figure 3. Upper panel: Brain regions in which a searchlight (cube with 7 voxels length – 343 voxels in total) achieved above-chance classification accuracy of sounds in the blind group. Bottom panel: regions in which classification accuracy achieved by the searchlight was different in the blind and the sighted. Warm colours indicate higher decoding accuracy in blind participants whereas cold colours represent higher decoding accuracy in sighted participants. Data for sighted participants were taken from [S1]. The colored overlays represent the lateral occipitotemporal complex (LOTC, green), the parahippocampal place area (PPA, red) and the fusiform face area (FFA, blue). The ROIs for these areas were defined as spheres (radius = 10 mm) centered around Talairach coordinates reported in the previous studies (LOC – [S2]; PPA – [S3]; FFA – [S4]). These ROIs were then projected onto a cortical surface**.** Threshold: $p < 0.005$ voxel-wise, familywise-error corrected for multiple comparisons across whole brain using cluster extent. Statistical significance of the results was assessed using permutation procedure (10 000 permutations, with 2 FWHM variance smoothing).

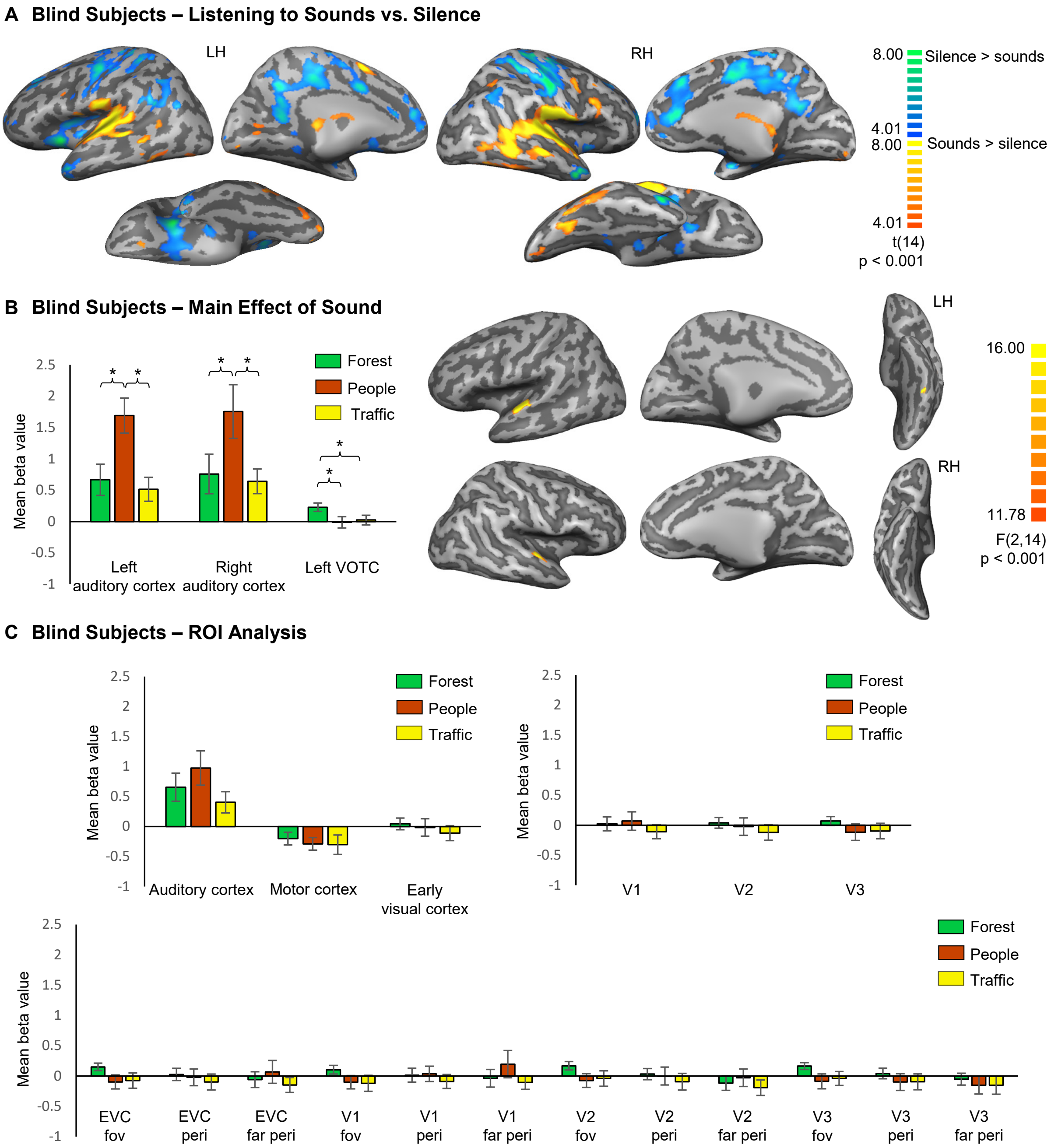

Figure S3. Results of the univariate analysis. Related to Figure 1. (A) A comparison of activations induced by listening to sounds and by periods of silence (apart from MRI scanner noise). Warm colours represent stronger activation for sounds. Cold colours – stronger activation during periods of silence. (B) Brain areas in which a significant main effect of sound was detected. A barplot represents mean beta value for each sound condition, relative to the periods of silence, within clusters identified as significant by a whole-brain F-test. (C) Mean beta values for each sound condition, relative to the periods of silence, in ROIs that were used in the multivariate analysis reported in the main text. Note that no significant between-sound differences in the univariate response were observed. Statistical thresholds: (A-B) Whole brain results were thresholded at $p < 0.001$ voxel-wise, corrected for multiple comparisons using cluster extent. (B-C) Within-ROI differences between sounds were tested with two-tailed Wilcoxon tests, Bonferroni-corrected for three comparisons within each ROI. * $p < 0.05$. Error bars indicate SEM.

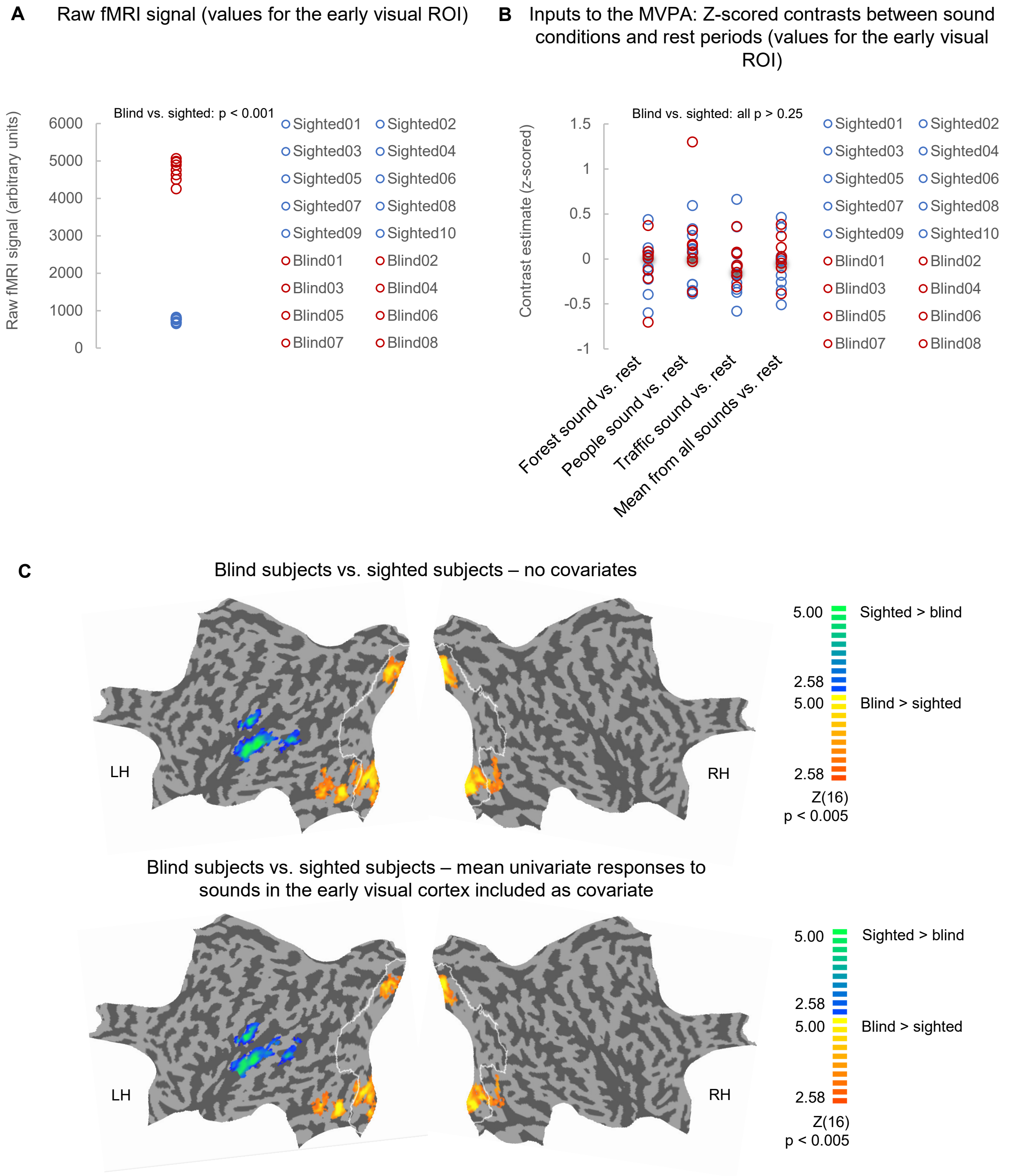

Figure S4. Control analyses exploring potential influence of different scanners on the between-group comparisons. Related to Figure 4. (A) fMRI signal in the early visual cortex, averaged across all volumes and runs for each participant. The early visual cortex ROI was defined as a cluster in which higher decoding accuracy in blind participants was observed in the searchlight analysis reported in the main text. (B) fMRI signal in the early visual cortex ROI after normalization procedure performed before the multivariate analysis: z-scored contrast estimates (sound > rest) for each sound and their mean, averaged across all voxels within the early visual cortex ROI, presented for each participant. Note that there are no detectable between-group differences in the univariate signal in the early visual cortex ROI after normalization. (C) Between-group comparisons of decoding accuracies obtained in the searchlight analysis, performed without any covariate (upper panel) and with individual means of univariate responses to sounds in the early visual ROI as a second-level covariate

(lower panel). Threshold: $p < 0.005$ voxel-wise, familywise-error corrected for multiple comparisons across whole brain using cluster extent. Statistical significance of the results was assessed using permutation procedure (10 000 permutations, with 2 FWHM variance smoothing).

	EVC	EVC fovea	EVC periphery	EVC far periphery
Blind subject 1	0.001	0.653	0.004	0.001
Blind subject 2	0.014	0.063	0.364	0.003
Blind subject 3	0.166	0.89	0.659	0.023
Blind subject 4	0.026	0.078	0.024	0.012
Blind subject 5	0.001	0.005	0.004	0.017
Blind subject 6	0.106	0.62	0.103	0.046
Blind subject 7	0.001	0.35	0.011	0.006
Blind subject 8	0.023	0.378	0.601	0.053
**Count of subjects with p < 0.05**	**6**	**1**	**4**	**7**

Table S1. Individual p-values. Related to Figure 2. For each subject, actual classification accuracy obtained for a given ROI was compared with accuracies obtained in 1000 permutations with labels randomly assigned. EVC – early visual cortex (i.e., areas V1, V2 and V3 combined).

ROI	Mean difference	Mean difference - lower 95% CI	Mean difference - upper 95% CI	p-value	FDR-corrected p-value
**EVC**	**0.34**	**0.24**	**0.44**	**0.003**	**0.010**
**V1**	**0.11**	**0.02**	**0.20**	**0.038**	**0.047**
**V2**	**0.20**	**0.10**	**0.31**	**0.010**	**0.017**
**V3**	**0.35**	**0.29**	**0.41**	**0.000**	**0.001**
EVC fovea	0.09	-0.07	0.25	0.171	0.171
**EVC periphery**	**0.22**	**0.11**	**0.33**	**0.007**	**0.014**
**EVC far periphery**	**0.31**	**0.11**	**0.48**	**0.011**	**0.017**
V1 fovea	0.07	-0.01	0.13	0.097	0.108
**V1 periphery**	**0.16**	**0.07**	**0.24**	**0.007**	**0.014**
**V1 far periphery**	**0.32**	**0.19**	**0.44**	**0.002**	**0.010**
**V2 fovea**	**0.14**	**0.06**	**0.24**	**0.033**	**0.045**
V2 periphery	0.11	0.00	0.24	0.101	0.108
**V2 far periphery**	**0.23**	**0.16**	**0.30**	**0.002**	**0.010**
**V3 fovea**	**0.31**	**0.16**	**0.44**	**0.011**	**0.017**
**V3 periphery**	**0.26**	**0.16**	**0.36**	**0.004**	**0.012**
**V3 far periphery**	**0.24**	**0.17**	**0.33**	**0.003**	**0.010**

Table S2. Testing the sound decoding accuracy in the early visual cortex of blind participants against chance level – the results of the bootstrapping procedure. Related to Figure 1. False

discover rate correction was applied across all tests performed to account for multiple comparisons. Significant results are bolded. CI – confidence interval.

Supplemental References

S1. Vetter, P., Smith, F.W., and Muckli, L. (2014). Decoding sound and imagery content in early visual cortex. Curr. Biol. 24, 1256–1262.

S2. He, C., Peelen, M. V, Han, Z., Lin, N., Caramazza, A., and Bi, Y. (2013). Selectivity for large nonmanipulable objects in scene-selective visual cortex does not require visual experience. Neuroimage 79, 1–9.

S3. Peelen, M. V., Bracci, S., Lu, X., He, C., Caramazza, A., and Bi, Y. (2013). Tool Selectivity in Left Occipitotemporal Cortex Develops without Vision. J. Cogn. Neurosci. 25, 1225–1234.

S4. Kanwisher, N., McDermott, J., & Chun, M. M. (1997). The fusiform face area: a module in human extrastriate cortex specialized for face perception. Journal of Neuroscience, 17(11), 4302-4311.
