# Supplementary material for: Decoding Natural Sounds in Early “Visual” Cortex of Congenitally Blind Individuals
Source: Curr Biol. 2020 Aug 3;30(15):3039–3044.e2. doi: 10.1016/j.cub.2020.05.071 (PMC7416107; doi:10.1016/j.cub.2020.05.071)
Supplement: Document S2. Article plus Supplemental Information [file mmc2.pdf]

Report

Current Biology

Decoding Natural Sounds in Early "Visual" Cortex of Congenitally Blind Individuals

Highlights

- Sounds can be decoded from early visual cortex activity in blind individuals
- Sound decoding accuracy increases from foveal to peripheral early visual regions
- Visual imagery is not necessary for auditory feedback to early visual cortex
- Early visual cortex organization by eccentricity develops without visual experience

Authors

Petra Vetter, Łukasz Bola, Lior Reich, Matthew Bennett, Lars Muckli, Amir Amedi

Correspondence

petra.vetter@rhul.ac.uk, petra.vetter@unifr.ch

In Brief

Natural sounds can be distinguished based on early visual cortex activity in sighted people. Is this effect driven by visual imagery? Vetter et al. report successful sound decoding, increasing from fovea to periphery, in people blind from birth, proving that visual imagery is not necessary for sound representation in these early visual areas.

Vetter et al., 2020, Current Biology *30*, 3039–3044
August 3, 2020 © 2020 The Authors. Published by Elsevier Inc.
https://doi.org/10.1016/j.cub.2020.05.071

CellPress

Current Biology

CellPress
OPEN ACCESS

Report

Decoding Natural Sounds in Early "Visual" Cortex of Congenitally Blind Individuals

Petra Vetter,[1,7,9,*] Łukasz Bola,[2,3,7] Lior Reich,[4] Matthew Bennett,[5] Lars Muckli,[5,8] and Amir Amedi[4,6,8]
[1]Department of Psychology, Royal Holloway, University of London, Egham Hill, Egham, Surrey TW20 0EX, UK
[2]Institute of Psychology, Jagiellonian University, ul. Ingardena 6, 30-060 Kraków, Poland
[3]Department of Psychology, Harvard University, William James Hall, 33 Kirkland Street, Cambridge, MA 02138, USA
[4]Department of Medical Neurobiology, Faculty of Medicine, Hebrew University Jerusalem, Ein Kerem, PO Box 12271, Jerusalem 91120, Israel
[5]Institute of Neuroscience and Psychology, University of Glasgow, 62 Hillhead Street, Glasgow G12 8QB, UK
[6]The Baruch Ivcher Institute for Brain, Cognition & Technology, The Baruch Ivcher School of Psychology, Interdisciplinary Center Herzliya, Reichman University, PO Box 167, Herzliya 461010, Israel
[7]These authors contributed equally
[8]These authors contributed equally
[9]Lead Contact
*Correspondence: petra.vetter@rhul.ac.uk or petra.vetter@unifr.ch
https://doi.org/10.1016/j.cub.2020.05.071

SUMMARY

Complex natural sounds, such as bird singing, people talking, or traffic noise, induce decodable fMRI activation patterns in early visual cortex of sighted blindfolded participants [1]. That is, early visual cortex receives non-visual and potentially predictive information from audition. However, it is unclear whether the transfer of auditory information to early visual areas is an epiphenomenon of visual imagery or, alternatively, whether it is driven by mechanisms independent from visual experience. Here, we show that we can decode natural sounds from activity patterns in early "visual" areas of congenitally blind individuals who lack visual imagery. Thus, visual imagery is not a prerequisite of auditory feedback to early visual cortex. Furthermore, the spatial pattern of sound decoding accuracy in early visual cortex was remarkably similar in blind and sighted individuals, with an increasing decoding accuracy gradient from foveal to peripheral regions. This suggests that the typical organization by eccentricity of early visual cortex develops for auditory feedback, even in the lifelong absence of vision. The same feedback to early visual cortex might support visual perception in the sighted [1] and drive the recruitment of this area for non-visual functions in blind individuals [2, 3].

RESULTS

Decoding of Natural Sounds in Congenitally Blind Individuals

To investigate the presence of sound representation in early visual cortex of blind individuals, we acquired fMRI data from 8 congenitally blind participants listening attentively to natural sounds. We used three natural sounds (one exemplar each): a forest scene (bird singing and a stream); a crowd scene (people talking without clear semantic information); and a street scene (traffic noise with cars and motorbikes; Figure 1A). We derived boundaries of early visual areas (V1, V2, and V3) and their foveal and peripheral regions, using cortex-based alignment and overlaying probabilistic retinotopic maps from sighted participants onto reconstructed brain surfaces of blind participants (Figure 1B). Using multi-variate pattern analysis (MVPA), we decoded the three different sounds from fMRI activity patterns in the corresponding early "visual" areas of blind individuals [1, 4, 5] (STAR Methods).

We found that we can decode natural sounds significantly above chance in all early visual areas (all early visual areas [EVCs] together, V1, V2, and V3) in the congenitally blind group (Figure 1C; all $p = 0.001$ from permutation testing), with remarkable consistency across individual participants (Figure 2). Confusion matrices illustrate similar classifier's predictions for each of the three sounds included in the experiment (Figure S1). Unsurprisingly, sound decoding worked very well in auditory cortex (positive control) but at chance level in motor cortex (negative control; Figure 1C). The successful decoding of sounds in early visual areas mirrors the results previously found in a group of sighted participants (Figure 1D; data taken from [1]). In order to investigate the role of visual imagery in the transfer of auditory information to early visual cortex, we previously conducted a series of control experiments in the sighted, concluding that visual imagery is unlikely to fully explain the observed pattern of activity in early visual areas [1]. However, we also concluded that the influence of visual imagery on auditory-induced activation patterns in visual cortex could not be ruled out entirely. Given that all blind participants lacked sight from birth on, and therefore lacked visual imagery, our current results in the blind group demonstrate that visual imagery is not a prerequisite of auditory feedback to early visual cortex.

Next, we looked at the eccentricity pattern of sound decoding accuracy in early visual cortex. We found that, in early visual cortex of blind individuals, sound decoding accuracy increased from foveal to peripheral regions, as indicated by a significant

This is an open access article under the CC BY license (http://creativecommons.org/licenses/by/4.0/).

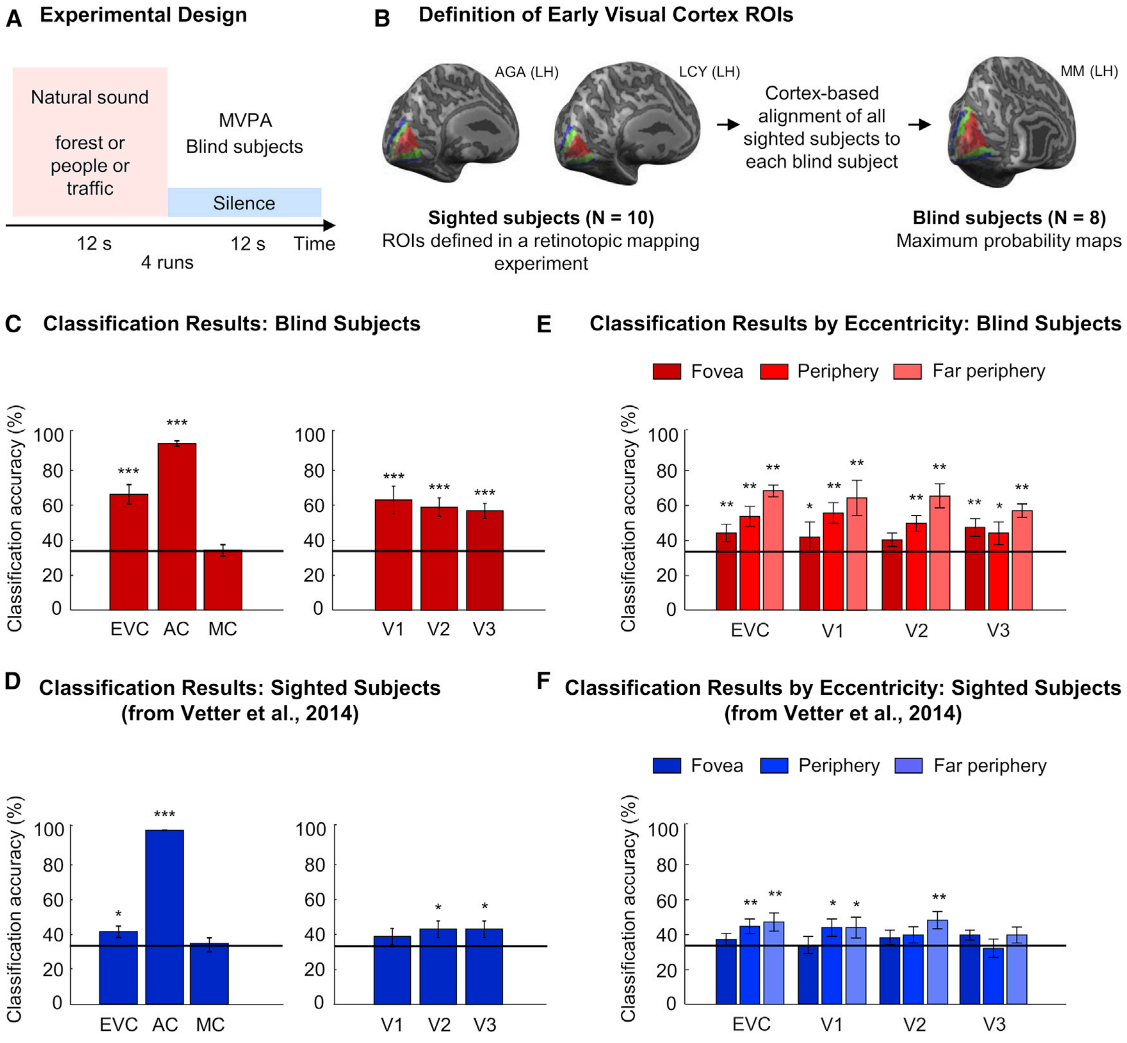

Figure 1. Experimental Design and Classification Results

(A) Congenitally blind individuals (n = 8) participated in an fMRI experiment in which they were listening to three natural sounds interleaved with silent periods (apart from MRI scanner noise). Multi-voxel pattern analysis (MVPA) was used to decode the sounds from participants' early visual cortex activity patterns.

(B) In a separate fMRI session, retinotopic mapping was performed for a group of sighted participants to individually define early visual areas V1, V2, and V3. These retinotopically defined regions of interest (ROIs) were then mapped onto a cortical reconstruction of each blind participant using cortex-based alignment. Aligned ROIs were converted into maximum probability maps that were then used in the data analysis as early visual ROIs for blind participants. Additionally, auditory cortex and motor cortex ROIs were defined for each participant using brain atlases.

(C and D) Mean classification accuracy of the classifier distinguishing the three natural sounds in the different ROIs in (C) blind participants and (D) sighted participants. The data for sighted participants (n = 10) were taken from [1] (the results for auditory cortex and motor cortex were recalculated within the same ROIs that were used for blind participants). Early visual cortex (EVC) contains V1, V2, and V3. AC, auditory cortex; MC, motor cortex. Chance level (one out of three) is marked with a black line. Error bars indicate SEM. Testing against chance level was performed with a permutation analysis. Results for V1, V2, and V3 were corrected for multiple comparisons, within each group, with a single threshold test. *$p < 0.05$; **$p < 0.01$; ***$p = 0.001$.

(E and F) Mean classification accuracies for all visual ROIs divided into three eccentricities (fovea, periphery, and far periphery) in both (E) the blind and (F) sighted group. Results were corrected for multiple comparisons within each group using the false discovery rate. *$p < 0.05$; **$p < 0.01$.

See also Figures S1 and S3 and Table S2.

eccentricity effect in an eccentricity by area ANOVA (Figure 1E; main effect of eccentricity: $F(2,14) = 9.69$, $p = 0.002$, partial eta squared = 0.58; all other ANOVA effects: $p > 0.25$) and a significant linear contrast for the eccentricity factor ($F(1,7) = 14.72$; $p = 0.006$; partial eta squared = 0.68). The observed gradient of decoding accuracy is comparable to the one previously found for sighted individuals (Figure 1F; data taken from [1]). Thus, a spatial pattern of auditory feedback modulation of early visual cortex activity is present in the blind group and similar to the one found in the sighted group. This suggests that the typical organization of early visual areas by eccentricity develops even despite the life-long absence of vision.

A whole-brain searchlight analysis in the blind group (Figure 3) revealed above-chance sound decoding in multisensory areas, such as superior temporal sulcus and middle temporal gyrus, similarly as shown previously in sighted participants [1]. This

CellPress
OPEN ACCESS

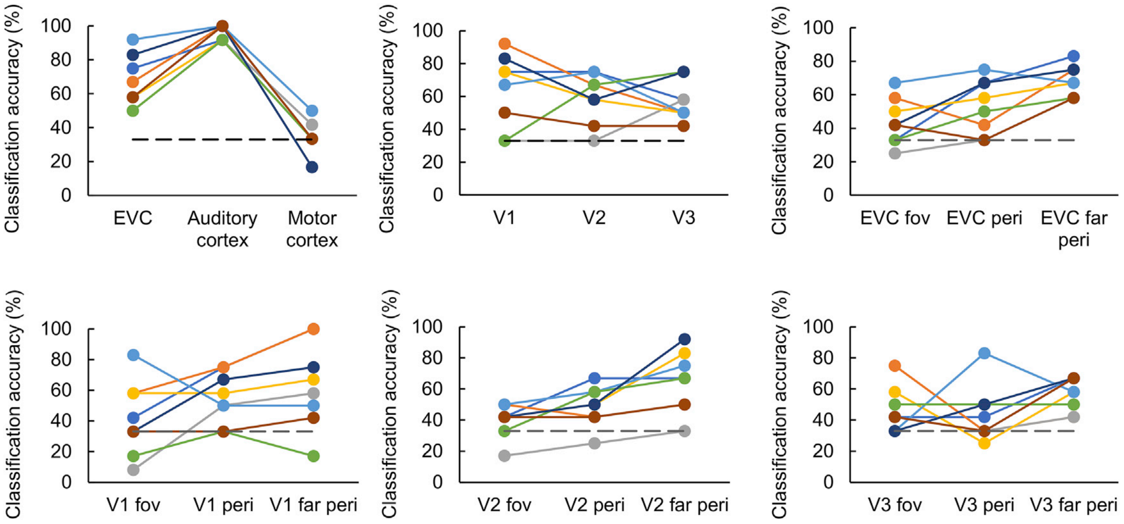

Figure 2. Classification Results for Individual Blind Participants

Chance level (one out of three) is marked with dashed lines. EVC, early visual cortex (areas V1, V2, and V3 combined). See also Table S1.

suggests that sound-related information is represented in largely the same cortical network in both populations. When we assessed the significance of the searchlight results in a non-parametric group model, which allows the use of a more sensitive voxelwise statistical threshold than parametric models [6], we also detected above-chance decoding of sounds in ventral visual stream areas of the blind group, e.g., the lateral occipital complex (LOC), parahippocampal place area (PPA), and fusiform face area (FFA) (Figure S2). The sounds used in this experiment convey different kinds of categorical information, e.g., they are animate or inanimate. Thus, this result is in line with previous findings of preserved category preference in the ventral visual stream areas in congenitally blind individuals [7–11].

On the univariate level (Figure S3), none of the three sounds elicited substantial blood-oxygen-level-dependent (BOLD) response for the contrast sound > rest in early visual areas, and most importantly, no sound elicited a higher or lower univariate response than another (see region of interest [ROI] analysis presented in Figure S3C: Wilcoxon tests, all $p > 0.1$; similar effects in the sighted, see Supplemental Information of [1]). Therefore, all decoding effects from the MVPA were driven by small, subthreshold activity differences across voxels in each ROI.

Sound Decoding in the Blind Group and the Sighted Group—Direct Comparisons and Control Analyses

Overall, our results indicate a high level of correspondence between cortical organization for sound processing in congenitally blind (the current study) and sighted individuals [1]. This correspondence was observed despite the dramatic between-group difference in sensory experience and the fact that both groups were scanned using different MRI scanners (STAR Methods). However, the qualitative comparison of the results for both groups also hints at certain between-group differences, for example, higher decoding accuracy for early visual cortex of the blind group, relative to decoding accuracy obtained for the same region in the sighted group (Figures 1C and 1D). Given that MVPA is typically performed on data that are normalized separately for each participant (see STAR Methods for details on the normalization procedure), which makes MVPA relatively invariant to differences in raw MRI signal that might arise from using different MRI scanners, we decided to supplement the main within-subject findings, reported above, with direct between-group comparisons.

In line with the pattern visible in qualitative comparisons, overall sound decoding accuracy in early visual cortex was significantly higher in the blind group than in the sighted group (62% versus 42%; Mann-Whitney $U = 7$; $p = 0.002$). A group × area × eccentricity ANOVA revealed a main effect of group ($F(1,16) = 10.00$; $p = 0.006$; partial eta squared = 0.39) and a main effect of eccentricity ($F(2,32) = 8.62$; $p = 0.001$; partial eta squared = 0.35), as well as a significant linear contrast for the eccentricity factor ($F(1,16) = 14.22$; $p = 0.002$; partial eta squared = 0.47). However, no group × eccentricity interaction was detected ($F(2,32) = 1.89$, $p = 0.168$, partial eta squared = 0.11; all other ANOVA effects: $p > 0.08$). Interestingly, in auditory cortex, we observed a trend toward lower decoding accuracy in the blind than in the sighted group (Mann-Whitney $U = 20$; $p = 0.083$), hinting at the possibility of the shift in distribution of sound representation, across auditory and visual cortices, in the blind group. To directly examine this possibility, we tested for the interaction between the group (blind participants and sighted participants) and the sensory region (early visual cortex and auditory cortex) in sound decoding accuracy. Because the data obtained for auditory cortex were not suitable for parametric ANOVA, due to ceiling effects (Figures 1C and 1D), we performed a non-parametric interaction test: first, for each participant, we calculated the difference between decoding accuracy for auditory cortex and early visual cortex; subsequently, these difference scores were compared between groups using the Mann-Whitney test. The comparison was highly significant ($U = 2.5$; $p < 0.001$). This result indicates that visual deprivation might result in a sound representation that is more distributed across these two sensory regions [2, 12, 13]. As expected, we did not detect a between-group difference in decoding accuracy

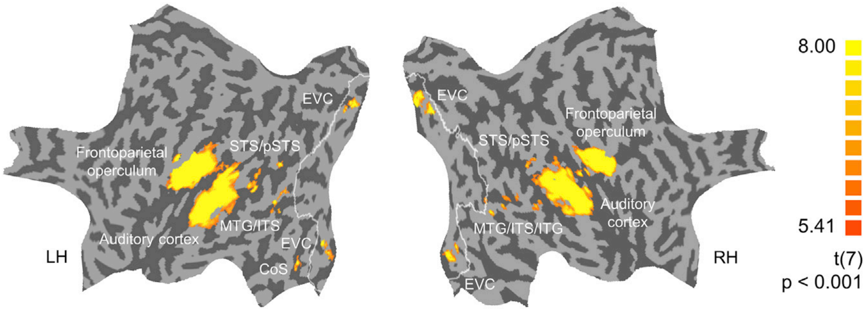

Figure 3. Results of the Whole-Brain Searchlight Analysis in the Blind Group

Brain regions in which a searchlight (cube with 7 voxels length—343 voxels in total) achieved above-chance classification accuracy of sounds in the blind group. Threshold: $p < 0.001$ voxelwise, corrected for multiple comparisons using cluster extent. The searchlight analysis was performed in the volume space, and results are displayed on a standard flattened cortical surface reconstruction for visualization purposes. White outlines represent combined Brodmann areas 17 and 18, as defined by a BrainVoyager brain atlas. CoS, collateral sulcus; EVC, early visual cortex; ITG, inferior temporal gyrus; ITS, inferior temporal sulcus; MTG, middle temporal gyrus; pSTS, posterior superior temporal sulcus; STS, superior temporal sulcus. See also Figure S2.

for the motor cortex (Mann-Whitney U = 38.5; $p > 0.25$). These effects were confirmed in the whole-brain between-group comparison of searchlight results (Figure 4), in which significant results were detected only in visual cortex (higher decoding accuracy in the blind group) and in auditory cortex (higher decoding accuracy in the sighted group); no significant group effects were detected outside these two sensory regions, neither in the motor cortex nor in multisensory regions.

The spatial specificity of the observed between-group effects, as well as their opposite direction in visual and auditory cortices, suggest that these effects were not driven by different MRI scanners, as this would have most likely manifested itself in a more general way. Nevertheless, as a control analysis, we empirically investigated the characteristics of the BOLD signal in the early visual ROI that showed significantly better decoding in the blind group than in the sighted group in the searchlight analysis (Figure S4). As expected, the raw BOLD signal values obtained for both groups were clearly different, which is likely to be an effect of different MRI scanners (Figure S4A). However, no between-group differences were observed in the values of normalized, *Z* scored contrast estimates (sound > rest) that served as input into the MVPA analyses, for neither of the three sounds nor their mean (Figure S4B; Mann-Whitney tests, all $p > 0.25$). These results suggest that the normalization procedure applied to the data before MVPA decoding was successful and that the observed group differences in decoding accuracy were driven by sounds eliciting spatial activity patterns being distinguishable better or worse in one group than another rather than by global differences in BOLD signal. To illustrate this point further, we included individual means of the univariate responses to sounds in the early visual ROI as a covariate in the whole-brain between-group comparison of decoding accuracies obtained in the searchlight analysis. The between-group difference in visual and auditory cortices replicated, even with this covariate (Figure S4C). Thus, even when we regressed out potential residual differences in input values to the MVPA analysis that might have arisen due to different scanners (or other methodological issues), we still found robust between-group differences for early visual cortex and for auditory cortex. In summary, our control analyses suggest that the observed between-group effects arise from the subtle differences in activation patterns and not from a global difference in signal due to different MRI scanners.

Note that all within-subject effects, especially the successful sound decoding in early visual cortex and the fovea-periphery eccentricity effects, are independent of direct group comparisons and were confirmed in each group independently. One of the potential limitations of the within-subject comparisons is that the blind group is relatively small due to challenges related to finding congenitally blind participants with no or minimal light perception (STAR Methods). Nevertheless, individual data show that the within-subject group effects are remarkably consistent across blind participants (Figure 2). Indeed, the main effects reported in our study—that is, the successful sound decoding in early visual cortex of the blind individuals, driven primarily by the decoding in the peripheries of this region—are clearly visible even at the level of individual p values, computed separately for each subject and ROI (Table S1).

To further confirm that the reported effects were not driven by outliers, we compared the decoding accuracies obtained for early visual areas of blind individuals to chance level using a bootstrapping procedure (STAR Methods). Because in this procedure various subsets of participants are included in a sample considered in each iteration (i.e., during each iteration, participants are drawn to a sample randomly, with replacements), results are unlikely to be affected by outliers. In this analysis, we largely replicated the results presented in Figure 1 (Table S2). Furthermore, to confirm that the fovea-periphery gradient effect is robust, we entered decoding accuracies for each of the eccentricities in the blind group (fovea, peripheries, and far peripheries; V1, V2, and V3 were combined for each eccentricity) into a non-parametric counterpart of a one-way repeated-measure ANOVA (the Friedman test). In line with the results of the parametric analysis, we observed a significant effect of eccentricity (chi-square = 9.48; $p = 0.009$). Overall, in combination with individual data, these control analyses show that our results in the blind group are robust and not driven by outliers.

DISCUSSION

Our study shows that natural sounds can be decoded successfully in early visual areas of congenitally blind individuals. Therefore, neither visual experience nor visual imagery is necessary for a transfer of sound-related information to early visual cortex. In fact, were visual imagery critical for this auditory feedback, it

CellPress
OPEN ACCESS

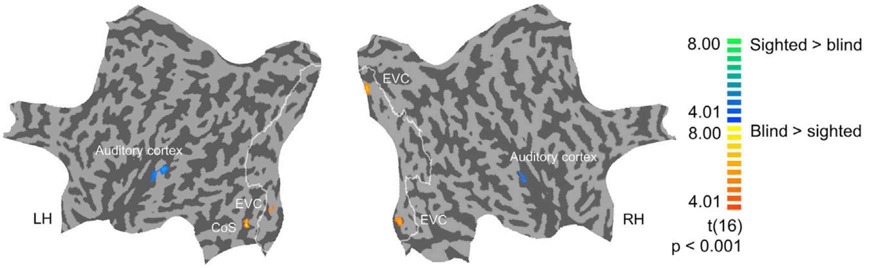

Figure 4. Results of the Whole-Brain Searchlight Analysis: Comparison between Blind and Sighted Participants

Regions in which classification accuracy achieved by the searchlight (cube with 7 voxels length—343 voxels in total) was different in the blind and the sighted. Warm-color hues indicate higher decoding accuracy in blind participants, whereas cold-color cues represent higher decoding accuracy in sighted participants. Data for sighted participants were taken from [1]. Threshold: $p < 0.001$ voxel-wise, corrected for multiple comparisons using cluster extent. The searchlight analysis was performed in the volume space, and results are displayed on a standard flattened cortical surface reconstruction for visualization purposes. White outlines represent combined Brodmann areas 17 and 18, as defined by a BrainVoyager brain atlas. CoS, collateral sulcus; EVC, early visual cortex. See also Figure S4.

should have boosted decoding accuracy in the sighted and lack of visual imagery should have diminished decoding accuracy in the blind group. Our results show that this was not the case, suggesting that, even in the sighted, sound decoding is not purely driven by visual imagery.

We also found that auditory feedback follows the organization of early visual cortex along an eccentricity gradient in the blind group, similarly as previously documented in the sighted [1]. This result suggests that the typical organization by eccentricity of early visual cortex can, to a large extent, develop even without any visual experience. Our finding complements previous results demonstrating retinotopic-like organization of functional connectivity of early visual cortex in blind individuals, highly similar to the ones observed in the sighted [14]. Higher sound decoding accuracy in peripheral areas of early visual cortex in both populations is also in line with previous evidence that, relative to the foveal part, the periphery has richer connections with numerous non-visual regions, particularly with higher level auditory areas and multisensory regions in the temporal and the parietal lobe [15–18]. Our results indicate that at least some of these connections might be preserved and functional, even in the lifelong absence of vision.

Sounds did not elicit differential univariate BOLD responses in early visual cortex, neither in the blind nor in the sighted population. This precludes the possibility that sound decoding worked on the basis of sounds attracting differential levels of attention or arousal, as attention strongly modulates univariate BOLD responses in early visual cortex [19, 20]. All sounds were normalized for amplitude, and in early visual cortex of sighted individuals, sound decoding was successful even when the classifier was trained and tested on activity patterns elicited by sounds that differ in basic auditory features, as long as categorical distinctions between sounds were the same in the training and the testing set (e.g., "animate" versus "inanimate"; see [1]). This generalization would not have worked if individual sound exemplars were distinguished only based on low-level acoustic (or visual) features. Instead, this result suggests that information reaching early visual cortex of sighted participants is, to an extent, content specific and possibly semantic. Given the high degree of correspondence between the results from sighted and blind participants, it is conceivable that the successful decoding in blind individuals may rely on the same type of high-level inputs.

In the sighted, auditory feedback to early visual cortex might be relevant for visual perception, in line with predictive coding theories [21]. In blind individuals, the same inputs might drive the recruitment of early visual areas for higher cognitive functions, such as verbal memory, language processing, or numerical computations [2, 3, 13]. A testable hypothesis is that this kind of recruitment could be most pronounced in peripheral parts of early visual cortex of blind individuals, as peripheral areas are primary receivers of auditory and, potentially, high-level information [15, 16]. The foveal part of early visual cortex of blind individuals may, in turn, retain its typical preference for high-resolution spatial processing, such as recognizing Braille characters or localizing sound in space—tasks that are known to involve early visual cortex in both sighted and blind individuals [2, 22–27]. In summary, our results add to the growing body of evidence that early visual cortex might support other functions than purely feedforward processing, in the absence and presence of visual input.

STAR★METHODS

Detailed methods are provided in the online version of this paper and include the following:

- KEY RESOURCES TABLE
- RESOURCE AVAILABILITY
  - Lead Contact
  - Materials Availability
  - Data and Code Availability
- EXPERIMENTAL MODEL AND SUBJECT DETAILS
- METHOD DETAILS
  - Stimuli and experimental procedures
  - Data collection
- QUANTIFICATION AND STATISTICAL ANALYSIS

SUPPLEMENTAL INFORMATION

Supplemental Information can be found online at https://doi.org/10.1016/j.cub.2020.05.071.

ACKNOWLEDGMENTS

This work was supported by a Daniel Turnberg Travel Fellowship, Academy of Medical Sciences, United Kingdom, to P.V., a National Science Centre Poland doctoral scholarship (2017/24/T/HS6/00367) and a Polish Ministry of Science grant (DN/MOB/023/V/2017) to Ł.B., a European Union's Horizon 2020 Framework Programme for Research and Innovation under the specific grant agreement nos. 785907 and 945539 (Human Brain Project SGA2 and SGA3) to L.M., an ERC Consolidator Grant (773121), a James S. McDonnell Foundation, United States, scholar award (no. 652 220020284), and a Joy ventures grant to A.A. We thank Ella Striem-Amit for helpful discussions and Lucy S. Petro and Stephanie Badde for comments on the manuscript.

AUTHOR CONTRIBUTIONS

P.V., L.M., and A.A. conceived the study; P.V. and L.R. collected the data; Ł.B., P.V., and M.B. analyzed the data; P.V. and Ł.B. wrote and revised the manuscript; and L.M. and A.A. provided funding and contributed to interpretation and manuscript writing.

DECLARATIONS OF INTERESTS

The authors declare no competing interests.

Received: July 5, 2019
Revised: February 12, 2020
Accepted: May 21, 2020
Published: June 18, 2020

REFERENCES

1. Vetter, P., Smith, F.W., and Muckli, L. (2014). Decoding sound and imagery content in early visual cortex. Curr. Biol. *24*, 1256–1262.
2. Amedi, A., Raz, N., Pianka, P., Malach, R., and Zohary, E. (2003). Early 'visual' cortex activation correlates with superior verbal memory performance in the blind. Nat. Neurosci. *6*, 758–766.
3. Bedny, M. (2017). Evidence from blindness for a cognitively pluripotent cortex. Trends Cogn. Sci. *21*, 637–648.
4. Smith, F.W., and Muckli, L. (2010). Nonstimulated early visual areas carry information about surrounding context. Proc. Natl. Acad. Sci. USA *107*, 20099–20103.
5. Haxby, J.V., Connolly, A.C., and Guntupalli, J.S. (2014). Decoding neural representational spaces using multivariate pattern analysis. Annu. Rev. Neurosci. *37*, 435–456.
6. Eklund, A., Nichols, T.E., and Knutsson, H. (2016). Cluster failure: why fMRI inferences for spatial extent have inflated false-positive rates. Proc. Natl. Acad. Sci. USA *113*, 7900–7905.
7. van den Hurk, J., Van Baelen, M., and Op de Beeck, H.P. (2017). Development of visual category selectivity in ventral visual cortex does not require visual experience. Proc. Natl. Acad. Sci. USA *114*, E4501–E4510.
8. Wang, X., Peelen, M.V., Han, Z., He, C., Caramazza, A., and Bi, Y. (2015). How visual is the visual cortex? Comparing connectional and functional fingerprints between congenitally blind and sighted individuals. J. Neurosci. *35*, 12545–12559.
9. Mahon, B.Z., Anzellotti, S., Schwarzbach, J., Zampini, M., and Caramazza, A. (2009). Category-specific organization in the human brain does not require visual experience. Neuron *63*, 397–405.
10. He, C., Peelen, M.V., Han, Z., Lin, N., Caramazza, A., and Bi, Y. (2013). Selectivity for large nonmanipulable objects in scene-selective visual cortex does not require visual experience. Neuroimage *79*, 1–9.
11. Peelen, M.V., Bracci, S., Lu, X., He, C., Caramazza, A., and Bi, Y. (2013). Tool selectivity in left occipitotemporal cortex develops without vision. J. Cogn. Neurosci. *25*, 1225–1234.
12. Dormal, G., Rezk, M., Yakobov, E., Lepore, F., and Collignon, O. (2016). Auditory motion in the sighted and blind: early visual deprivation triggers a large-scale imbalance between auditory and "visual" brain regions. Neuroimage *134*, 630–644.
13. Amedi, A., Floel, A., Knecht, S., Zohary, E., and Cohen, L.G. (2004). Transcranial magnetic stimulation of the occipital pole interferes with verbal processing in blind subjects. Nat. Neurosci. *7*, 1266–1270.
14. Striem-Amit, E., Ovadia-Caro, S., Caramazza, A., Margulies, D.S., Villringer, A., and Amedi, A. (2015). Functional connectivity of visual cortex in the blind follows retinotopic organization principles. Brain *138*, 1679–1695.
15. Falchier, A., Clavagnier, S., Barone, P., and Kennedy, H. (2002). Anatomical evidence of multimodal integration in primate striate cortex. J. Neurosci. *22*, 5749–5759.
16. Rockland, K.S., and Ojima, H. (2003). Multisensory convergence in calcarine visual areas in macaque monkey. Int. J. Psychophysiol. *50*, 19–26.
17. Eckert, M.A., Kamdar, N.V., Chang, C.E., Beckmann, C.F., Greicius, M.D., and Menon, V. (2008). A cross-modal system linking primary auditory and visual cortices: evidence from intrinsic fMRI connectivity analysis. Hum. Brain Mapp. *29*, 848–857.
18. Beer, A.L., Plank, T., and Greenlee, M.W. (2011). Diffusion tensor imaging shows white matter tracts between human auditory and visual cortex. Exp. Brain Res. *213*, 299–308.
19. Watanabe, M., Cheng, K., Murayama, Y., Ueno, K., Asamizuya, T., Tanaka, K., and Logothetis, N. (2011). Attention but not awareness modulates the BOLD signal in the human V1 during binocular suppression. Science *334*, 829–831.
20. Gandhi, S.P., Heeger, D.J., and Boynton, G.M. (1999). Spatial attention affects brain activity in human primary visual cortex. Proc. Natl. Acad. Sci. USA *96*, 3314–3319.
21. Keller, G.B., and Mrsic-Flogel, T.D. (2018). Predictive processing: a canonical cortical computation. Neuron *100*, 424–435.
22. Campus, C., Sandini, G., Concetta Morrone, M., and Gori, M. (2017). Spatial localization of sound elicits early responses from occipital visual cortex in humans. Sci. Rep. *7*, 10415.
23. Norman, L.J., and Thaler, L. (2019). Retinotopic-like maps of spatial sound in primary 'visual' cortex of blind human echolocators. Proc. Biol. Sci. *286*, 20191910.
24. Bola, Ł., Matuszewski, J., Szczepanik, M., Droździel, D., Sliwinska, M.W., Paplińska, M., Jednoróg, K., Szwed, M., and Marchewka, A. (2019). Functional hierarchy for tactile processing in the visual cortex of sighted adults. Neuroimage *202*, 116084.
25. Sadato, N., Pascual-Leone, A., Grafman, J., Ibañez, V., Deiber, M.P., Dold, G., and Hallett, M. (1996). Activation of the primary visual cortex by Braille reading in blind subjects. Nature *380*, 526–528.
26. Cohen, L.G., Celnik, P., Pascual-Leone, A., Corwell, B., Falz, L., Dambrosia, J., Honda, M., Sadato, N., Gerloff, C., Catalá, M.D., and Hallett, M. (1997). Functional relevance of cross-modal plasticity in blind humans. Nature *389*, 180–183.
27. Petro, L.S., Paton, A.T., and Muckli, L. (2017). Contextual modulation of primary visual cortex by auditory signals. Philos. Trans. R. Soc. Lond. B Biol. Sci. *372*, 20160104.
28. Nichols, T.E., and Holmes, A.P. (2002). Nonparametric permutation tests for functional neuroimaging: a primer with examples. Hum. Brain Mapp. *15*, 1–25.
29. Chang, C.C., and Lin, C.J. (2001). LIBSVM—a library for support vector machines. http://www.csie.ntu.edu.tw/~cjlin/libsvm.
30. Pereira, F., and Botvinick, M. (2011). Information mapping with pattern classifiers: a comparative study. Neuroimage *56*, 476–496.
31. Wandell, B.A., Dumoulin, S.O., and Brewer, A.A. (2007). Visual field maps in human cortex. Neuron *56*, 366–383.
32. Schira, M.M., Tyler, C.W., Breakspear, M., and Spehar, B. (2009). The foveal confluence in human visual cortex. J. Neurosci. *29*, 9050–9058.
33. Benson, N.C., Butt, O.H., Datta, R., Radoeva, P.D., Brainard, D.H., and Aguirre, G.K. (2012). The retinotopic organization of striate cortex is well predicted by surface topology. Curr. Biol. *22*, 2081–2085.
34. Stelzer, J., Chen, Y., and Turner, R. (2013). Statistical inference and multiple testing correction in classification-based multi-voxel pattern analysis (MVPA): random permutations and cluster size control. Neuroimage *65*, 69–82.

CellPress
OPEN ACCESS

STAR★METHODS

KEY RESOURCES TABLE

REAGENT or RESOURCE	SOURCE	IDENTIFIER
Deposited Data		
MRI data	Openneuro.org	https://doi.org/10.18112/openneuro.ds002715.v1.0.0; RRID: SCR_005031
Experimental code and sound data files	Github.com	https://github.com/Muckli-lab/NaturalSound-Stimulation; RRID: SCR_002630
MVPA analysis code	Github.com	https://github.com/Muckli-lab/MVP-analysis-tool-box; RRID: SCR_002630
MRI data	Ebrains.eu	https://kg.ebrains.eu/search/instances/Dataset/4f6e1509-2e7f-44dd-a45c-c100cd7728a3; RRID: SCR_017612
Software and Algorithms		
Presentation 13	Neurobehavioral Systems	https://www.neurobs.com; RRID: SCR_002521
BrainVoyager 20.6	BrainInnovation, Maastricht	http://www.brainvoyager.comRRID: SCR_013057
MATLAB 2018a	MathWorks	https://www.mathworks.com; RRID: SCR_001622
IBM SPSS 26	IBM Corp, Armonk, NY	https://www.ibm.com/analytics/spss-statistics-software; RRID: SCR_002865
SnPM toolbox (v. 13.1.08)	[28]	http://www.nisox.org/Software/SnPM13/; RRID: SCR_002092
LIBSVM toolbox (v. 3.23)	[29]	http://www.csie.ntu.edu.tw/~cjlin/libsvm; RRID: SCR_010243
SearchMight toolbox (Linux_x86_64.0.2.5)	[30]	http://www.franciscopereira.org/searchmight/
NeuroElf (v. 1.1 7251)	Neuroelf.net	https://neuroelf.net/; RRID: SCR_014147

RESOURCE AVAILABILITY

Lead Contact

For further information and requests for resources, Lead Contact will be Petra Vetter (petra.vetter@rhul.ac.uk; petra.vetter@unifr.ch).

Materials Availability

This study did not generate new materials.

Data and Code Availability

The MRI dataset generated during this study is available on openneuro.org under https://doi.org/10.18112/openneuro.ds002715.v1.0.0. The code generated to run the experiment is available on https://github.com/Muckli-lab/NaturalSound-Stimulation and to perform the MVPA analysis is available on https://github.com/Muckli-lab/MVP-analysis-tool-box. The MRI dataset is also accessible on the EBRAINS platform of the Human Brain Project under https://kg.ebrains.eu/search/instances/Dataset/4f6e1509-2e7f-44dd-a45c-c100cd7728a3.

EXPERIMENTAL MODEL AND SUBJECT DETAILS

Eight congenitally blind individuals with intact hearing (5 females, mean age 33.4 years, range 23-39 years, 4 left handers, mean education duration 13.6 years, range 12-17 years) participated in the study. Reasons for blindness were: microphthalmia in three participants of which one also had retinal detachment, retinopathy of prematurity in four participants, and enophthalmos in one participant. One blind participant had very faint light perception, all others had no light perception at all. All participants received detailed information of the study, signed informed consent and were paid for their participation. The study was approved by the Tel-Aviv Sourasky Medical Centre Ethics Committee, Israel.

Data from healthy participants with intact vision and hearing (n = 10, 7 females, mean age 24.1 years, range 20-33 years) were used as controls and taken from Experiment 1 of Vetter et al. [1], see Supplemental Online Information for details [1].

METHOD DETAILS

Stimuli and experimental procedures

Stimuli and experimental procedures of the study with blind individuals were the same as in [1], Experiment 1 unless reported in the following. In brief, participants listened to one exemplar each of three natural sounds, traffic noise (a busy road with cars and motorbikes), a forest scene (birds singing and a stream) and a crowd scene (people talking in a foreign language without clear semantic

information). All sounds were downloaded from https://www.soundsnap.com, normalized for amplitude (volume) and presented mono, using Presentation (Neurobehavioral Systems). Seven out of eight blind participants listened to sounds cut to 12 s with an ISI of 12 s, one blind participant listened to a shorter version of sounds of 6 s and ISI of 6 s. The sighted participants all listened to the long version (12 s sound, 12 s ISI). Each sound was repeated 6 times per run (in a pseudo-randomized order such that never two of the same sounds were repeated one after the other), resulting in 222 volumes (117 in the shorter version). All participants completed 4 runs. All participants were familiarised with the sounds before the start of the experiment to ensure they recognized each sound correctly. There was no specific behavioral task, but participants were instructed to listen to the sounds attentively throughout the experiment.

Data collection

Blood oxygen level dependent signals in the blind group were acquired in a 3 T General Electric MRI scanner (TR = 2 s, TE = 30 ms, Resolution: 3.2 × 3.2 × 2.5 mm voxels, 35 slices, flip angle: 77°, iPAT factor = 2). BOLD signals in the sighted group were acquired in a 3 T Siemens Tim Trio MRI scanner (TR = 2 s, TE = 30 ms, resolution 2.5 × 2.5 × 2.5 mm, 35 slices, flip angle 77°, iPAT factor 2; see supplemental information of [1]).

QUANTIFICATION AND STATISTICAL ANALYSIS

Data were analyzed with BrainVoyager 20.6 (BrainInnovation, Maastricht) with standard preprocessing (including slice scan time correction, 3D rigid body motion correction, temporal high-pass filter, no spatial smoothing for the multivariate pattern analysis, and 6 mm FWHM spatial smoothing for univariate analysis presented in Figure S2). Retinotopic maps [31, 32] acquired in all sighted participants for the study described in [1] were mapped onto a cortical surface reconstruction of each blind participant, using the cortex-based alignment procedure [33]. Aligned early visual ROIs were converted into maximum probability maps, which were then used in the data analysis as early visual ROIs for blind participants. Auditory cortex and motor cortex ROIs were defined using BrainVoyager cortical atlases, which were cortex-based aligned to the cortical surface of each blind participant. Auditory cortex ROI was created by combining Brodmann areas 41, 42 and 22 together. Hand motor area was chosen as the motor cortex ROI.

Single block beta weights were estimated for all surface vertices of each ROI during natural sound stimulation (versus rest periods) and fed into a linear support vector machine classification algorithm (LIBSVM toolbox [29]). Beta values were normalized by z-scoring in the training dataset and the same normalization was applied for the testing data. Classification was performed one-versus-one for each of the three combinations of sounds and results were averaged. ROIs were combined across both hemispheres. The classifier was trained on 3 runs to distinguish between the three types of sounds and tested on the remaining 4th run in a leave-one-run-out cross-validation procedure (results were averaged across different folds of training and test dataset assignments). To determine statistical significance, a permutation analysis was performed, which included training and testing the classifier across 1000 permutations with randomized sound labels in each participant and each ROI. On the single subject level, p values were derived as the probability of getting a classification accuracy value as large as the real label performance in the randomization distribution [1, 28, 34]. On the group level, p values were derived using the same logic, from the mean randomization distribution and the mean real label performance, calculated for each ROI, across participants in a group. Additionally, to verify whether the results are robust to outlying values, testing against chance was also performed using bootstrapping procedure (number of samples = 10 000), as implemented in SPSS 26 (IBM Corp, Armonk, NY).

Testing for between-group differences in classification accuracy in the EVC, auditory cortex and the motor cortex was performed with a Mann-Whitney test. Results for V1, V2 and V3 within each group of participants were corrected for multiple comparisons with a single threshold test [28]. Results for all visual ROIs divided into three eccentricities (fovea, periphery, and far periphery) were FDR-corrected within each group of participants. Whole brain searchlight analyses were performed on the voxel level with the SearchMight toolbox [30] using a linear SVM. Group results were thresholded at $p < 0.001$ voxel-wise and corrected for multiple comparisons using cluster extent. The size of a cluster necessary to achieve correction at $p < 0.05$ was determined using Brain Voyager's Cluster-Level Statistical Threshold Estimator plugin. Additionally, to test for robustness of our results and to visualize less focal effects, a second group model was created using a nonparametric, permutation approach, as implemented in SnPM 13, http://nisox.org/Software/SnPM13/ [28]. The use of a non-parametric approach allows the use of more sensitive voxel-wise thresholds while keeping the false positive rate under strict control [6]. The non-parametric model was thresholded at $p < 0.005$ voxel-wise, familywise-error corrected for multiple comparisons using cluster extent. The significance of the results was determined with 10,000 permutations and 2 mm FWHM variance smoothing.

Current Biology, Volume *30*

Supplemental Information

Decoding Natural Sounds in Early "Visual" Cortex of Congenitally Blind Individuals

Petra Vetter, Łukasz Bola, Lior Reich, Matthew Bennett, Lars Muckli, and Amir Amedi

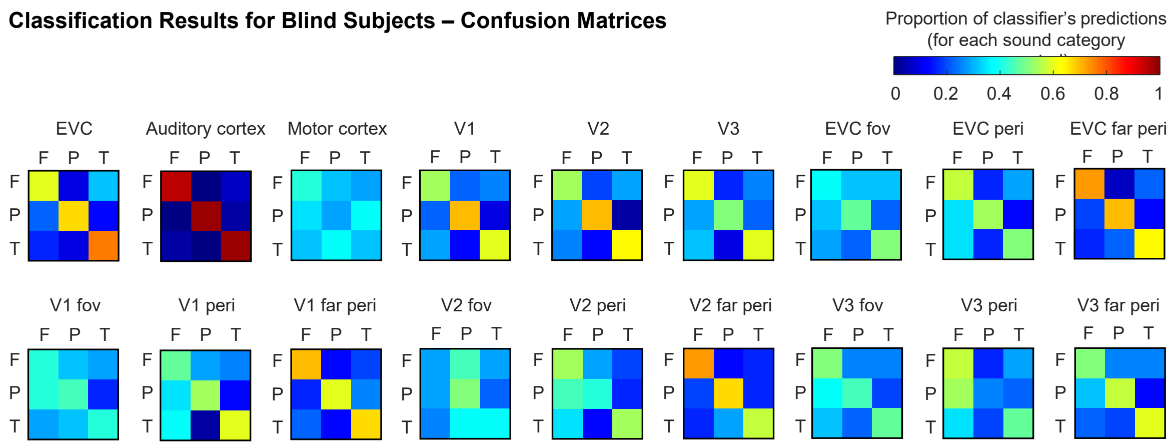

Figure S1. Confusion matrices representing classifier predictions for each sound condition in the group of blind participants. Related to Figure 1. Columns of the confusion matrices indicate the sound displayed (F, forest; P, people; T, traffic), and rows indicate classifier's predictions for this sound. The classifier's predictions for each sound are represented by colour hues, with warmer colours for higher proportion of predictions and colder colours for lower proportion of predictions. Note that in the ROIs in which above-chance classification accuracy was observed, the classifier was generally able to differentiate between all three sounds. EVC – early visual cortex.

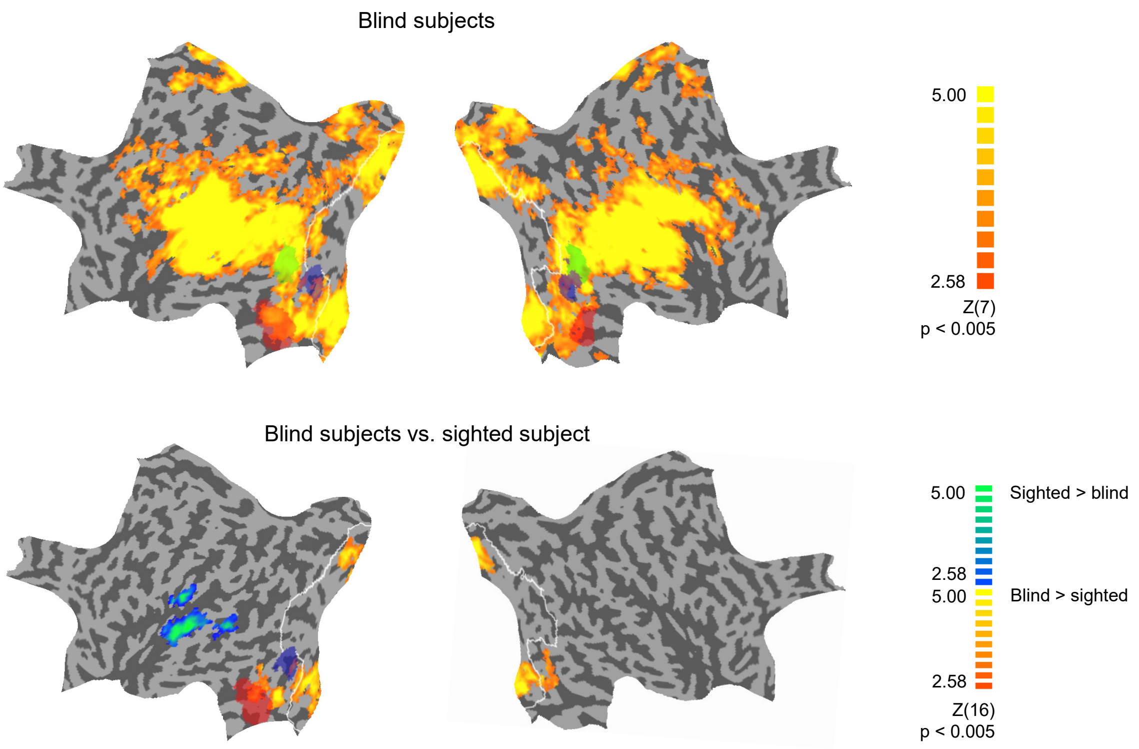

Figure S2. Results of the whole-brain searchlight analysis within a non-parametric model. Related to Figure 3. Upper panel: Brain regions in which a searchlight (cube with 7 voxels length – 343 voxels in total) achieved above-chance classification accuracy of sounds in the blind group. Bottom panel: regions in which classification accuracy achieved by the searchlight was different in the blind and the sighted. Warm colours indicate higher decoding accuracy in blind participants whereas cold colours represent higher decoding accuracy in sighted participants. Data for sighted participants were taken from [S1]. The colored overlays represent the lateral occipitotemporal complex (LOTC, green), the parahippocampal place area (PPA, red) and the fusiform face area (FFA, blue). The ROIs for these areas were defined as spheres (radius = 10 mm) centered around Talairach coordinates reported in the previous studies (LOC – [S2]; PPA – [S3]; FFA – [S4]). These ROIs were then projected onto a cortical surface**.** Threshold: $p < 0.005$ voxel-wise, familywise-error corrected for multiple comparisons across whole brain using cluster extent. Statistical significance of the results was assessed using permutation procedure (10 000 permutations, with 2 FWHM variance smoothing).

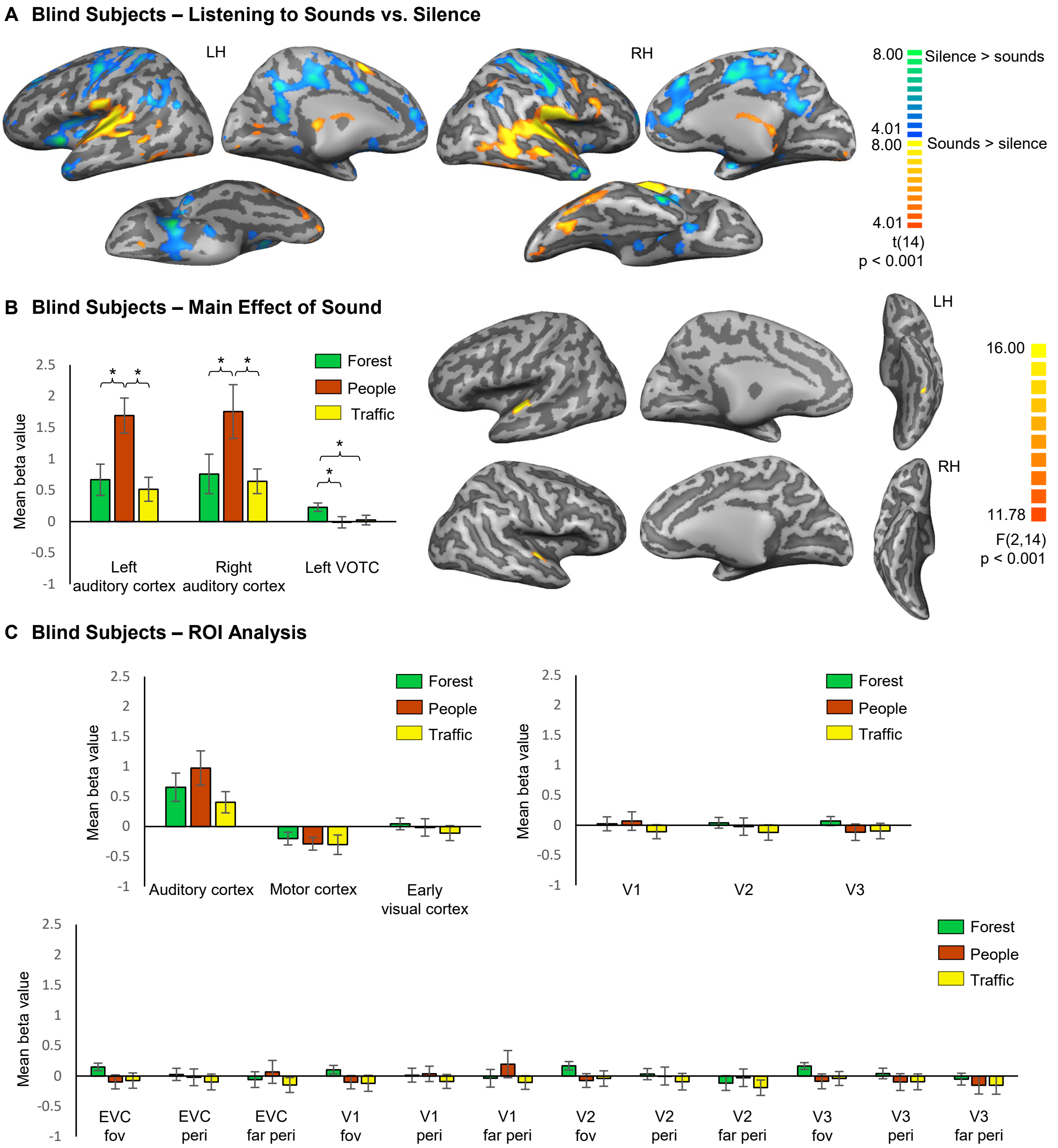

Figure S3. Results of the univariate analysis. Related to Figure 1. (A) A comparison of activations induced by listening to sounds and by periods of silence (apart from MRI scanner noise). Warm colours represent stronger activation for sounds. Cold colours – stronger activation during periods of silence. (B) Brain areas in which a significant main effect of sound was detected. A barplot represents mean beta value for each sound condition, relative to the periods of silence, within clusters identified as significant by a whole-brain F-test. (C) Mean beta values for each sound condition, relative to the periods of silence, in ROIs that were used in the multivariate analysis reported in the main text. Note that no significant between-sound differences in the univariate response were observed. Statistical thresholds: (A-B) Whole brain results were thresholded at $p < 0.001$ voxel-wise, corrected for multiple comparisons using cluster extent. (B-C) Within-ROI differences between sounds were tested with two-tailed Wilcoxon tests, Bonferroni-corrected for three comparisons within each ROI. * $p < 0.05$. Error bars indicate SEM.

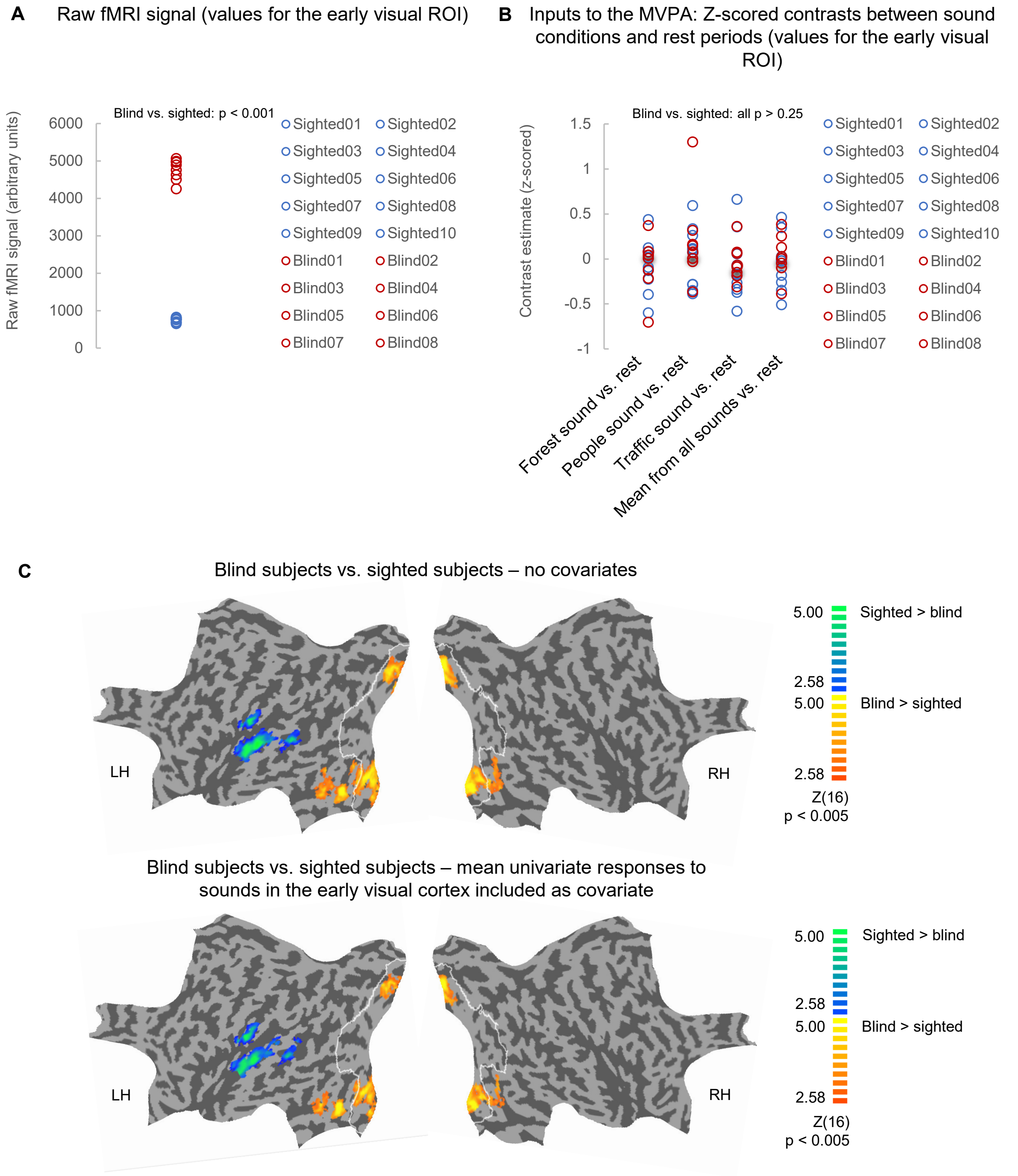

Figure S4. Control analyses exploring potential influence of different scanners on the between-group comparisons. Related to Figure 4. (A) fMRI signal in the early visual cortex, averaged across all volumes and runs for each participant. The early visual cortex ROI was defined as a cluster in which higher decoding accuracy in blind participants was observed in the searchlight analysis reported in the main text. (B) fMRI signal in the early visual cortex ROI after normalization procedure performed before the multivariate analysis: z-scored contrast estimates (sound > rest) for each sound and their mean, averaged across all voxels within the early visual cortex ROI, presented for each participant. Note that there are no detectable between-group differences in the univariate signal in the early visual cortex ROI after normalization. (C) Between-group comparisons of decoding accuracies obtained in the searchlight analysis, performed without any covariate (upper panel) and with individual means of univariate responses to sounds in the early visual ROI as a second-level covariate

(lower panel). Threshold: $p < 0.005$ voxel-wise, familywise-error corrected for multiple comparisons across whole brain using cluster extent. Statistical significance of the results was assessed using permutation procedure (10 000 permutations, with 2 FWHM variance smoothing).

	EVC	EVC fovea	EVC periphery	EVC far periphery
Blind subject 1	0.001	0.653	0.004	0.001
Blind subject 2	0.014	0.063	0.364	0.003
Blind subject 3	0.166	0.89	0.659	0.023
Blind subject 4	0.026	0.078	0.024	0.012
Blind subject 5	0.001	0.005	0.004	0.017
Blind subject 6	0.106	0.62	0.103	0.046
Blind subject 7	0.001	0.35	0.011	0.006
Blind subject 8	0.023	0.378	0.601	0.053
**Count of subjects with p < 0.05**	**6**	**1**	**4**	**7**

Table S1. Individual p-values. Related to Figure 2. For each subject, actual classification accuracy obtained for a given ROI was compared with accuracies obtained in 1000 permutations with labels randomly assigned. EVC – early visual cortex (i.e., areas V1, V2 and V3 combined).

ROI	Mean difference	Mean difference - lower 95% CI	Mean difference - upper 95% CI	p-value	FDR-corrected p-value
**EVC**	**0.34**	**0.24**	**0.44**	**0.003**	**0.010**
**V1**	**0.11**	**0.02**	**0.20**	**0.038**	**0.047**
**V2**	**0.20**	**0.10**	**0.31**	**0.010**	**0.017**
**V3**	**0.35**	**0.29**	**0.41**	**0.000**	**0.001**
EVC fovea	0.09	-0.07	0.25	0.171	0.171
**EVC periphery**	**0.22**	**0.11**	**0.33**	**0.007**	**0.014**
**EVC far periphery**	**0.31**	**0.11**	**0.48**	**0.011**	**0.017**
V1 fovea	0.07	-0.01	0.13	0.097	0.108
**V1 periphery**	**0.16**	**0.07**	**0.24**	**0.007**	**0.014**
**V1 far periphery**	**0.32**	**0.19**	**0.44**	**0.002**	**0.010**
**V2 fovea**	**0.14**	**0.06**	**0.24**	**0.033**	**0.045**
V2 periphery	0.11	0.00	0.24	0.101	0.108
**V2 far periphery**	**0.23**	**0.16**	**0.30**	**0.002**	**0.010**
**V3 fovea**	**0.31**	**0.16**	**0.44**	**0.011**	**0.017**
**V3 periphery**	**0.26**	**0.16**	**0.36**	**0.004**	**0.012**
**V3 far periphery**	**0.24**	**0.17**	**0.33**	**0.003**	**0.010**

Table S2. Testing the sound decoding accuracy in the early visual cortex of blind participants against chance level – the results of the bootstrapping procedure. Related to Figure 1. False

discover rate correction was applied across all tests performed to account for multiple comparisons. Significant results are bolded. CI – confidence interval.

Supplemental References

S1. Vetter, P., Smith, F.W., and Muckli, L. (2014). Decoding sound and imagery content in early visual cortex. Curr. Biol. 24, 1256–1262.

S2. He, C., Peelen, M. V, Han, Z., Lin, N., Caramazza, A., and Bi, Y. (2013). Selectivity for large nonmanipulable objects in scene-selective visual cortex does not require visual experience. Neuroimage 79, 1–9.

S3. Peelen, M. V., Bracci, S., Lu, X., He, C., Caramazza, A., and Bi, Y. (2013). Tool Selectivity in Left Occipitotemporal Cortex Develops without Vision. J. Cogn. Neurosci. 25, 1225–1234.

S4. Kanwisher, N., McDermott, J., & Chun, M. M. (1997). The fusiform face area: a module in human extrastriate cortex specialized for face perception. Journal of Neuroscience, 17(11), 4302-4311.
